# Flap size as a confounding variable in flap perfusion measurement with the Oxygen-to-see (O2C) analysis system in microvascular head and neck reconstruction – a retrospective analysis

**DOI:** 10.1007/s00784-025-06468-1

**Published:** 2025-08-07

**Authors:** Mark Ooms, Philipp Winnand, Marius Heitzer, Anna Bock, Marie Sophie Katz, Johannes Bickenbach, Frank Hölzle, Ali Modabber

**Affiliations:** 1 Department of Oral and Maxillofacial Surgery, University Hospital RWTH Aachen, Pauwelsstraße 30, 52074 Aachen, Germany; 2 Department of Intensive Care Medicine, University Hospital RWTH Aachen, Pauwelsstraße 30, 52074 Aachen, Germany

**Keywords:** Microvascular head and neck reconstruction, Free flap, Flap perfusion monitoring, Oxygen-to-see analysis system, Flap size

## Abstract

**Objectives:**

The Oxygen-to-see (O2C) analysis system is used for flap monitoring based on predefined threshold values for flap perfusion. However, flap size may be a confounding variable. The aim of this study was to investigate the relationship between flap size and flap perfusion in microvascular head and neck reconstruction.

**Materials and methods:**

Flap perfusion values measured with the O2C analysis system between 2011 and 2020 in 252 patients undergoing microvascular head and neck reconstruction with a radial free forearm flap (RFFF) or anterolateral thigh flap (ALTF) were retrospectively analyzed. Intraoperative and postoperative flap blood flow, hemoglobin concentration, and hemoglobin oxygen saturation at 8- and 2-mm tissue depths were compared between small (≤ median flap size) and large flaps (> median flap size) for RFFFs and ALTFs separately.

**Results:**

Intraoperative and postoperative hemoglobin concentration at a 2-mm tissue depth differed between small and large ALTFs (65.0 arbitrary units [AU] vs. 51.0 AU, *p* = 0.007; and 51.5 AU vs. 39.0 AU, *p* = 0.019). Both differences persisted in multivariable analysis (*p* < 0.001 and *p* = 0.012). Other differences were not observed or did not persist in multivariable analysis for RFFFs and ALTFs (all *p* > 0.05).

**Conclusions:**

Microvascular free flap perfusion is not related to flap size in terms of flap blood flow and hemoglobin oxygen saturation. This underscores the validity of predefined absolute threshold values in the context of flap monitoring based on perfusion measurement with the O2C analysis system.

**Clinical relevance:**

Flap perfusion measurement with the Oxygen-to-see (O2C) analysis system can be used for flap monitoring of small and large flaps.

## Introduction

Microvascular free flaps are the standard in the reconstruction of complex head and neck defects, with superior results and high success rates [1, 2]. Nevertheless, flap failures continue to occur, with detrimental consequences for patients in terms of the need for secondary surgical interventions or permanent impairments [1, 3]. Because the time interval between the occurrence and correction of vascular flap compromise is inversely related to the success of flap salvage, postoperative flap monitoring is essential for the timely detection of vascular flap compromise [1, 4–6].

The Oxygen-2-see (O2C) analysis system (LEA Medizintechnik, Germany) is an objective and reliable method of flap monitoring based on the measurement of flap perfusion at tissue depths of 8- and 2-mm with surface probes [4, 7, 8]. Based on threshold values for flap perfusion parameters (i.e., absolute threshold values for blood flow and hemoglobin oxygen saturation and relative threshold values for hemoglobin concentration), it facilitates the timely detection of vascular flap compromise [7, 8]. The predefined threshold values account for different flap types, such as the radial free forearm flap (RFFF) and the anterolateral thigh flap (ALTF) [7, 8]. However, different flap sizes of RFFFs and ALTFs due to the different amount of tissue required depending on defect size, as well as differences in the perforator number of ALTFs due to the varying anatomy in the anterolateral thigh region, remain largely unconsidered [7–11]. And this, although some studies have shown that flap size and perforator number influence microvascular flap perfusion and flap perfusion parameters, respectively [12–17]. However, RFFFs and ALTFs were either not represented at all or only represented in small numbers in these studies [13, 14]. It remains unclear whether flap size and perforator number have an influence on flap perfusion, and they could be confounding variables in flap monitoring with the O2C analysis system, potentially affecting the validity of predefined threshold values for perfusion parameters and, thus, clinical decision-making [7, 8].

The study aimed to investigate the relationship between the flap size of RFFFs and ALTFs and the perforator number of ALTFs and flap perfusion parameters.

## Materials and methods

### Study population

This retrospective study used data collected for routine clinical purposes and was approved by the local ethics committee of the Medical Faculty RWTH Aachen University (EK 309 − 20).

The study population included 252 patients consecutively who underwent extraoral and intraoral microvascular reconstruction in the head and neck region in our Department of Oral and Maxillofacial Surgery between 2011 and 2020. The patient exclusion criteria were incomplete data and age under 18 years.

Surgery duration and flap ischemia duration were defined as the time interval between the first incision and the last suture and the time interval between the interruption of flap perfusion due to dissection of the flap pedicle at the donor site and the resumption of flap perfusion after the release of the anastomosis at the recipient site, respectively. Comorbidities, such as diabetes and arterial hypertension, were defined according to disease-specific guidelines, and smoking status was defined as actual or past daily smoking for a period of at least six months, according to commonly used recommendations [18]. Prior neck dissection or irradiation was defined as the previous anatomic dissection of the recipient vessel used for anastomosis in the context of neck dissection or the irradiation of the recipient vessel used for anastomosis in the context of neck irradiation, respectively. Flap revision was defined as the surgical revision of the anastomosis, with return to the operating room, and flap success was defined as negative if the flap was removed due to flap necrosis.

All surgical procedures were conducted under general anesthesia. The number of perforators in the ALTFs was determined intraoperatively by surgical dissection. The anastomoses were performed in an end-to-end fashion for arteries and an end-to-side or end-to-end fashion for veins. Postoperatively, patients remained in the intensive care unit at least until the next morning, with invasive mechanical ventilation, analgosedation, invasive arterial blood pressure monitoring, and blood pressure regulation via the central venous administration of norepinephrine (target systolic pressure above 125 mmHg).

### Flap perfusion measurement data

Flap perfusion values were based on measurements with the O2C analysis system (O2C Oxygen-to-see, LEA Medizintechnik, Giesen, Germany), which combines the techniques of laser Doppler spectroscopy and white light spectroscopy [7, 8]. In brief, the flap perfusion value for blood flow (arbitrary units [AU]) at 8- and 2-mm tissue depths was determined via laser Doppler spectroscopy (830 nm; 30 mW) and calculated by analyzing the Doppler shift of the laser light due to the erythrocyte movement as the product of erythrocyte quantity and erythrocyte velocity [7, 19]. The flap perfusion values for hemoglobin concentration (AU) and hemoglobin oxygen saturation (%) at 8- and 2-mm tissue depths were determined via white light spectroscopy (500–800 nm; 50 W) and calculated by analyzing the sum of the absorbances and the color change of the white light in comparison to reference hemoglobin spectra with known oxygen saturation, respectively [7, 19].

Measurements were performed intraoperatively after the release of the anastomosis in the operating room and postoperatively on the first postoperative morning in the intensive care unit for 10 s with a lead time of four seconds under ambient light compensation control, with the surface probe placed in the center of the dried skin portion of the flap in a sterile sheath.

### Statistical analysis

Patients were divided into groups according to flap size, which were defined separately for each flap type (i.e., RFFF and ALTF) as small given a skin portion area less than or equal to the median of all flaps (RFFF: ≤ 35 cm²; ALTF: ≤ 70 cm²) and as large given a skin portion area greater than the median of all flaps (RFFF: >35 cm²; ALTF: >70 cm²). In addition, patients were categorized into two groups according to their American Society of Anesthesiologists score (ASA > 2 or ASA ≤ 2). Group differences in baseline data were analyzed with the Chi-squared test, Fisher exact test, or Freeman Halton test for categorical data and with the Mann Whitney test for metric data. Group differences in flap perfusion values were analyzed with the Mann Whitney test for flap size (small flaps vs. large flaps) and with the Kruskal Wallis test for number of perforators (1 vs. 2 vs. 3). Significant differences between groups in flap perfusion values were analyzed using multiple linear regression models adjusted for flap location (extraoral vs. intraoral), mean arterial blood pressure (mmHg), and administered catecholamine dose (µg/min per kg) for RFFFs and using multiple linear regression models adjusted for flap location (extraoral vs. intraoral), surgery duration (min), prior neck dissection (yes vs. no), mean arterial blood pressure (mmHg), and administered catecholamine dose (µg/min per kg) for ALTFs. Values of *p* < 0.05 were considered statistically significant. The statistical analysis was performed using SPSS Version 28 (SPSS, IBM, New York, USA) and GraphPad Prism Version 4.0 (GraphPad Software, Boston, USA).

## Results

### Study population characteristics

The study included 252 patients (149 patients reconstructed with RFFFs and 103 patients reconstructed with ALTFs) (Table 1). In the group of patients reconstructed with RFFFs (77 patients with a small flap [flap size ≤ 35 cm²] and 72 patients with a large flap [flap size > 35 cm²]), there was a difference in terms of flap location (*p* < 0.001). In the group of patients reconstructed with ALTFs (54 patients with a small flap [flap size ≤ 70 cm²] and 49 patients with a large flap [flap size > 70 cm²]), there were differences in terms of flap location (*p* = 0.032), surgery duration (*p* = 0.005), and prior neck dissection (*p* = 0.047). For both flap types, the groups of patients reconstructed with small or large flaps did not differ in terms of flap survival or flap revision (RFFF: *p* = 0.497 and *p* = 1.000; ALTF: *p* = 0.224 and *p* = 0.224). Seven RFFFs and one ALTF were revised due to venous congestion, and one ALTF was revised due to arterial insufficiency.

**Table 1:** Study population characteristics

Variable	All (*n* = 252)	RFFF (*n* = 149)	ALTF (*n* = 103)
Sex *(n)*			
male	132 (52.4%)	76 (51.0%)	56 (54.4%)
female	120 (47.6%)	73 (49.0%)	47 (45.6%)
Age (years)	64.0 (19.0)	64.0 (18.0)	66.0 (20.0)
BMI (kg/m²)	24.4 (6.3)	25.0 (6.0)	23.4 (5.9)
ASA (n)			
1 + 2	141 (56.0%)	92 (61.7%)	49 (47.6%)
3 + 4	111 (44.0%)	57 (38.3%)	54 (52.4%)
Flap location (n)* **			
tongue	41 (16.3%)	32 (21.5%)	9 (8.7%)
floor of mouth	61 (24.2%)	39 (26.2%)	22 (21.4%)
mandible	50 (19.8%)	17 (11.4%)	33 (32.0%)
maxilla + hard palate	31 (12.3%)	20 (13.4%)	11 (10.7%)
cheek	24 (9.5%)	16 (10.7%)	8 (7.8%)
soft palate	14 (5.6%)	11 (7.4%)	3 (2.9%)
extraoral	31 (12.3%)	14 (9.4%)	17 (16.5%)
Arterial anastomosis recipient vessel *(n)*			
ECA	15 (6.0%)	4 (2.7%)	11 (10.7%)
FAA	99 (39.3%)	59 (39.6%)	40 (38.8%)
LIA	11 (4.4%)	4 (2.7%)	7 (6.8%)
STA	127 (50.4%)	82 (55.0%)	45 (43.7%)
Surgery duration (min) **	540.0 (170.0)	513.0 (169.0)	562.0 (160.0)
Flap ischemia duration (min)	105.5 (34.0)	108.0 (36.0)	102.0 (32.0)
Diabetes *(n)*			
no	214 (84.9%)	127 (85.2%)	87 (84.5%)
yes	38 (15.1%)	22 (14.8%)	16 (15.5%)
Arterial hypertension *(n)*			
no	163 (64.7%)	89 (59.7%)	74 (71.8%)
yes	89 (35.3%)	60 (40.3%)	29 (28.2%)
Smoking status *(n)*			
no	154 (61.1%)	92 (61.7%)	62 (60.2%)
yes	98 (38.9%)	57 (38.3%)	41 (39.8%)
Prior neck dissection *(n)* **			
no	201 (79.8%)	124 (83.2%)	77 (74.8%)
yes	51 (20.2%)	25 (16.8%)	26 (25.2%)
Prior neck irradiation *(n)*			
no	226 (89.7%)	138 (92.6%)	88 (85.4%)
yes	26 (10.3%)	11 (7.4%)	15 (14.6%)
Flap survival *(n)*			
no	4 (1.6%)	2 (1.3%)	2 (1.9%)
yes	248 (98.4%)	147 (98.7%)	101 (98.1%)
Flap revision *(n)*			
no	243 (96.4%)	142 (95.3%)	101 (98.1%)
yes	9 (3.6%)	7 (4.7%)	2 (1.9%)

Parameters are indicated as numbers (with percentage) for categorical data (sex, ASA, flap location, arterial anastomosis recipient vessel, diabetes, arterial hypertension, smoking status, prior neck dissection, prior neck irradiation, flap survival, flap revision) or median (with interquartile range) for metric data (age, BMI, surgery duration, flap ischemia duration) (separately described for all patients, patients reconstructed with a RFFF, and patients reconstructed with an ALTF). * *p* < 0.05 corresponding to testing for differences between groups with small RFFFs (≤ 35 cm²; *n* = 77) and large RFFFs (> 35 cm²; *n* = 72) and ** *p* < 0.05 corresponding to testing for differences between groups with small ALTFs (≤ 70 cm²; *n* = 54) and large ALTFs (> 70 cm²; *n* = 49) with chi-squared test (sex, ASA, diabetes, arterial hypertension, smoking status, prior neck dissection), Fisher’s exact test (prior neck irradiation, flap survival, flap revision), and Freeman Halton test (flap location, arterial anastomosis recipient vessel) for categorical data and Mann Whitney test (age, BMI, surgery duration, flap ischemia duration) for metric data; abbreviations: RFFF = radial free forearm flap, ALTF = anterolateral thigh flap, BMI = body mass index, ASA = American Society of Anesthesiologists score, ECA = external carotid artery, FAA = facial artery, LIA = lingual artery, STA = superior thyroid artery

### Comparison of flap perfusion parameters based on flap size

**Fig. 1:**
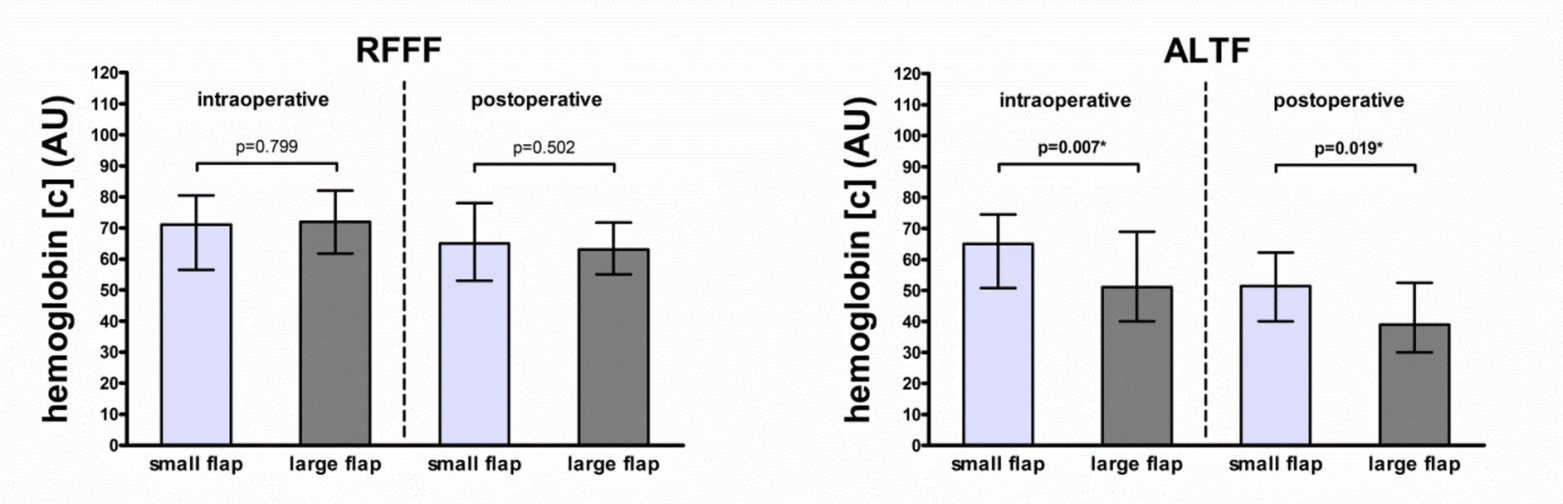
Comparison of hemoglobin concentration in 2-mm tissue depth. Box plot for intraoperative and postoperative hemoglobin concentration (AU) in 2-mm tissue depth for RFFFs (*n* = 149) (left) (small RFFFs (≤ 35 cm²; *n* = 77) and large RFFFs (> 35 cm²; *n* = 72)) and for ALTFs (*n* = 103) (right) (small ALTFs (≤ 70 cm²; *n* = 54) and large ALTFs (> 70 cm²; *n* = 49)); p-values corresponding to testing for differences between groups (small flaps vs. large flaps); significant p-values are bold (*p < 0.05 upon adjustment for prior neck dissection, surgery duration, flap location, mean arterial blood pressure (mmHg) and administered catecholamine dose (µg/min per kg) in multiple regression analysis); abbreviations: RFFF = radial free forearm flap, ATLF = anterolateral thigh flap, AU = arbitrary units

In ALTFs, postoperative blood flow at 8-mm tissue depth was increased in smaller flaps as compared to larger flaps (126.0 AU vs. 103.0 AU, *p* = 0.044), with the difference not persisting in multivariable testing (*p* = 0.377) (Table 2). In ALTFs, intraoperative and postoperative hemoglobin concentrations at 2-mm tissue depth were increased in small flaps as compared to large flaps (65.0 AU vs. 51.0 AU, *p* = 0.007; and 51.5 AU vs. 39.0 AU, *p* = 0.019, respectively), with both differences persisting in multivariable testing (*p* < 0.001 and *p* = 0.012) (Table 2; Fig. 1). In RFFFs, postoperative hemoglobin oxygen saturation at 8-mm tissue depth was increased in small flaps as compared to large flaps (70.0% vs. 62.5%, *p* = 0.039), without persistence in multivariable testing (*p* = 0.070) (Table 2). In ALTFs, postoperative hemoglobin oxygen saturation at 2-mm tissue depth was increased in smaller flaps as compared to larger flaps (59.0% vs. 49.0%, *p* = 0.030, respectively), with the difference not persisting in multivariable testing (*p* = 0.129).

**Table 2:** Flap perfusion parameter comparison between small and large flaps

Variable	RFFF (*n* = 149)			ALTF (*n* = 103)		
	Small (*n* = 77)	Large (*n* = 72)	*p*-value	Small (*n* = 54)	Large (*n* = 49)	*p*-value
Intraoperative measurement						
Flow (AU)						
8-mm	122.0 (70.5)	129.0 (74.3)	0.379	99.5 (76.5)	104.0 (70.0)	0.563
2-mm	29.0 (31.5)	28.5 (33.8)	0.661	21.5 (21.3)	18.0 (20.5)	0.887
Hemoglobin concentration (AU)						
8-mm	43.0 (18.0)	43.5 (18.8)	0.711	34.5 (15.5)	32.0 (18.0)	0.140
2-mm	71.0 (24.0)	72.0 (20.3)	0.799	65.0 (23.8)	51.0 (29.0)	0.007*
Hemoglobin oxygen saturation (%)						
8-mm	79.0 (27.5)	76.5 (28.3)	0.494	55.5 (32.8)	60.0 (29.0)	0.822
2-mm	81.0 (27.5)	79.0 (26.0)	0.142	65.5 (33.5)	60.0 (35.0)	0.309
Postoperative measurement						
Flow (AU)						
8-mm	132.0 (78.5)	127.5 (72.3)	0.915	126.0 (68.8)	103.0 (62.0)	**0.044**
2-mm	37.0 (41.0)	35.5 (34.5)	0.495	28.0 (29.5)	25.0 (25.5)	0.389
Hemoglobin concentration (AU)						
8-mm	43.0 (18.5)	39.5 (16.0)	0.632	31.0 (11.0)	31.0 (10.5)	0.691
2-mm	65.0 (25.0)	63.0 (16.8)	0.502	51.5 (22.3)	39.0 (22.5)	0.019*
Hemoglobin oxygen saturation (%)						
8-mm	70.0 (27.0)	62.5 (31.0)	**0.039**	47.0 (33.8)	64.0 (32.0)	0.059
2-mm	74.0 (26.5)	71.5 (21.8)	0.705	59.0 (27.3)	49.0 (38.5)	**0.030**

Parameters are indicated as median (with interquartile range) for intraoperative and postoperative measurement (separately described for patients reconstructed with RFFFs (subdivided in small RFFFs (≤ 35 cm²; *n* = 77) and large RFFFs (> 35 cm²; *n* = 72)) and patients reconstructed with ALTFs (subdivided in small ALTFs (≤ 70 cm²; *n* = 54) and large ALTFs (> 70 cm²; *n* = 49)); p-values corresponding to testing for differences between groups (small flaps vs. large flaps) with Mann Whitney test separately for RFFFs and ALTFs; significant p-values are bold (*p < 0.05 upon adjustment for prior neck dissection, surgery duration, flap location, mean arterial blood pressure (mmHg) and administered catecholamine dose (µg/min per kg) in multiple regression analysis); abbreviations: RFFF = radial free forearm flap, ATLF = anterolateral thigh flap, AU = arbitrary units

### Comparison of flap perfusion parameters based on perforator number

No differences were observed in flap perfusion parameters between ALTFs with one, two, or three perforators (all *p* > 0.05) (Table 3).

**Table 3:** Flap perfusion parameter comparison between perforator numbers

Variable	ALTF (*n* = 103)			
	1 perforator (*n* = 27)	2 perforators (*n* = 55)	3 perforators (*n* = 21)	*p*-value
Intraoperative measurement				
Flow (AU)				
8-mm	99.0 (73.0)	100.0 (75.0)	103.0 (89.5)	0.981
2-mm	22.0 (12.0)	19.0 (26.0)	18.0 (18.5)	0.940
Hemoglobin concentration (AU)				
8-mm	34.0 (15.0)	33.0 (17.0)	32.0 (13.5)	0.297
2-mm	64.0 (29.0)	57.0 (24.0)	68.0 (22.0)	0.087
Hemoglobin oxygen saturation (%)				
8-mm	68.0 (39.0)	57.0 (29.0)	62.0 (22.0)	0.723
2-mm	65.0 (43.0)	59.0 (33.0)	66.0 (23.5)	0.437
Postoperative measurement				
Flow (AU)				
8-mm	134.0 (76.0)	107.0 (53.0)	122.0 (91.0)	0.526
2-mm	18.0 (30.0)	28.0 (22.0)	31.0 (39.0)	0.298
Hemoglobin concentration (AU)				
8-mm	32.0 (11.0)	31.0 (8.0)	31.0 (15.0)	0.454
2-mm	43.0 (26.0)	44.0 (25.0)	46.0 (32.0)	0.981
Hemoglobin oxygen saturation (%)				
8-mm	52.0 (38.0)	58.0 (40.0)	52.0 (29.0)	0.997
2-mm	60.0 (32.0)	55.0 (31.0)	55.0 (33.0)	0.941

Parameters are indicated as median (with interquartile range) for intraoperative and postoperative measurement for patients reconstructed with ALTFs (subdivided in ALTFs with 1 perforator (*n* = 27), ALTFs with 2 perforators (*n* = 55), and ALTFs with 3 perforators (*n* = 21)); p-values corresponding to testing for differences between groups (flaps with 1 perforator vs. flaps with 2 perforators vs. flaps with 3 perforators) with Kruskal Wallis test; abbreviations: ATLF = anterolateral thigh flap, AU = arbitrary units

## Discussion

In this study, the influence of the flap size of RFFFs and ALTFs, as well as the perforator number of ALTFs, on flap perfusion was investigated, as these flap characteristics vary greatly, correlations between them and flap perfusion have been shown, and flap perfusion is used as a parameter in the context of flap monitoring [7–17].

Because the viability and, thus, survival of the microvascular free flap relies on sufficient perfusion, postoperative flap monitoring is crucial for the timely detection and correction of vascular flap compromise [4, 5, 7, 8]. For this purpose, the O2C analysis system is often used [7, 8]. It is based on the measurement of flap perfusion with respect to predefined threshold values indicating vascular flap compromise [7, 8]. The threshold values for the perfusion parameters are absolute for flap blood flow and hemoglobin oxygen saturation (RFFF/ALTF: flap blood flow < 20/15 AU [8-mm tissue depth] and < 10/5 AU [2-mm tissue depth]; hemoglobin oxygen saturation < 15/10% [8- and 2-mm tissue depth]), and relative for hemoglobin concentration (RFFF/ALTF: increase > 30/30% [8- and 2-mm tissue depth]) [7, 8]. However, these threshold values do not account for the different flap sizes of RFFFs and ALTFs or the different perforator number of ALTFs, although the influences of both flap characteristics on flap perfusion parameters have been found [12–17]. Still, such influences for RFFFs and ALTFs remain unclear, despite the fact that they may affect the validity of the predefined threshold values for flap monitoring with the O2C analysis system, particularly for absolute threshold values, and, thus, the decision to revise a flap [7, 8].

The aim of this study was therefore to investigate the relationship between flap perfusion parameters, as measured with the O2C analysis system, and flap size for RFFFs and ALTFs and the perforator number for ALTFs, as the determination of threshold values for flap sizes individually is hampered by the low flap revision rate overall [2, 20]. The classification of small and large flaps in terms of median flap size (small flap ≤ median; large flap > median) was chosen because no general definition of small and large RFFFs and ALTFs exists and to ensure approximately equal group sizes [10, 11].

This study showed that flap size has an influence on flap perfusion parameters in terms of a higher intraoperative and postoperative hemoglobin concentration at a 2-mm tissue depth in small ALTFs as compared to large ALTFs only, while other differences in ALTFs and RFFFs were not observed at all or did not persist in multivariable testing.

In general, the lack of differences between small and large flaps in terms of flap blood flow and hemoglobin oxygen saturation, both of which are usually positively correlated, was unexpected, as larger flaps should have higher blood flow due to lower vascular resistance [7, 12, 14, 17]. In addition, higher blood flow would have been expected in larger flaps given their higher metabolic demands [11, 14]. In the context of flap vasculature, the difference in hemoglobin concentration only in ALTFs could be due to the different vascular anatomy of the two flaps, as RFFFs typically have multiple perforators to perfuse a relatively small amount of tissue, with increasing cross-sectional area from the source vessel to the flap, acting more as a vascular shunt, whereas ALTFs typically have only one perforator or at least a few perforators to perfuse a relatively large amount of tissue with decreasing cross-sectional area from the source vessel to the flap, with the perforator vessel being the narrowest point in the flap vasculature [14, 21–26]. Thus, the increased hemoglobin concentration at a 2-mm tissue depth in small as compared to large ALTFs, despite similar flap blood flow values, may reflect higher flap flow resistance with the congestion of blood drainage, potentially due to a critically lower number of venous perforators in smaller ALTFs, which restricts venous outflow through the deep venous system, as the superficial venous system ceases to function after flap elevation with circumcision of the skin portion [14, 17, 27, 28]. However, these differences in hemoglobin concentration between small and large ALTFs have no clinical implications, as the threshold values for hemoglobin concentration are relative values that refer to previously measured values [7, 8].

This study also showed that the perforator number for ALTFs had no influence on flap perfusion parameters.

This is in line with an animal study showing that the values for the perfusion parameters of flap blood flow, hemoglobin concentration, and hemoglobin oxygen saturation measured with the O2C analysis system were similar between flaps with different perforator numbers [15]. Theoretically, flap blood flow is limited by the narrowest point of the flap vasculature (i.e., the perforator vessel), with the vessel flow resistance being related to the vessel radius raised to the fourth power [22, 23, 25]. Earlier studies have shown that free flaps with only one perforator had higher blood flow as compared to flaps with more than one perforator, which was attributed to lower total flow resistance with one perforator with a larger putative radius as compared to multiple perforators with smaller putative radii [16, 23, 25].

In general, the lack of differences between small and large flaps in terms of flap blood flow and hemoglobin oxygen saturation could be due to the regulation of flap perfusion in accordance with flap tissue requirements independent of the flap size [10, 11, 21, 29, 30]. This could explain the observed lack of association between flap size and flap failure in previous studies, as well as in the present study [10, 11, 29].

This study has limitations by measuring flap perfusion at only one flap spot (i.e., the center of the flap) and only at two timepoints (i.e., intraoperatively and once postoperatively). However, the data were analyzed in a retrospective manner without the chance to change the number of measurement spots. Moreover, additional data for presumably confounding variables (i.e., mean arterial blood pressure and catecholamine dose administered [potentially causing flap vessel vasoconstriction and perfusion reduction]) were only available for the two time points [31]. Furthermore, the influence of confounding factors, such as perfusion variability within the flap tissue, which is not covered by flap perfusion measurement at only one flap spot, and variations among patients in vascular anatomy in terms of vessel length and vessel diameter, with a potential influence on flap perfusion, cannot be excluded [23, 25]. However, the measurement of flap perfusion on one spot in the center of the flap is commonly performed for flap monitoring with the O2C analysis system [7, 8]. In addition, flap size was calculated only two-dimensionally, without considering flap thickness, although this could be mitigated by the groups being similar in terms of BMI, which correlates with flap thickness [11, 32]. In general, multiple regression analysis with adjustment for the administered catecholamine dose, among other potentially confounding variables, was performed only for significant differences in flap perfusion, so a lack of effect of flap size on flap perfusion due to the vasoconstrictive effect of catecholamines cannot be ruled out [31].

In conclusion, the flap perfusion parameters measured with the O2C analysis system for the purpose of flap monitoring are not influenced by flap size for RFFFs and ALTFs or the perforator number for ALTFs in terms of the absolute threshold values indicating vascular flap compromise (i.e., flap blood flow and hemoglobin oxygen saturation). With regard to clinical implications, these findings highlight the validity of these threshold values. Further studies are needed to confirm these results while controlling for potential confounding factors.

## Conclusion

The results of this study show that flap size for RFFFs and ALTFs and perforator number for ALTFs have no influence on flap perfusion parameters as measured with the O2C analysis system for the purpose of flap monitoring, specifically in terms of the threshold values for flap blood flow and hemoglobin oxygen saturation. This emphasizes the validity of the predefined threshold values indicating vascular flap compromise regardless of flap size for RFFFs and ALTFs and perforator numbers for ALTFs.

## Data Availability

The data underlying this article is available on reasonable request to the corresponding author.
